# Proteomic Analysis of the Extracellular Matrix Produced by Mesenchymal Stromal Cells: Implications for Cell Therapy Mechanism

**DOI:** 10.1371/journal.pone.0079283

**Published:** 2013-11-14

**Authors:** Adam Harvey, Ten-Yang Yen, Irina Aizman, Ciara Tate, Casey Case

**Affiliations:** 1 SanBio Inc., Mountain View, California, United States of America; 2 Department of Biology, San Francisco State University, San Francisco, California, United States of America; 3 Department of Chemistry and Biochemistry, San Francisco State University, San Francisco, California, United States of America; University of Pittsburgh, United States of America

## Abstract

Mesenchymal stromal cells (MSCs) transiently transfected with *notch1 intracellular domain* (*NICD*) are beneficial for neurological disorders as observed in several preclinical studies. Extracellular matrix (ECM) derived from *NICD*-transfected MSCs has been previously shown to support *in vitro* neural cell growth and survival better than that of un-transfected MSCs. To understand the underlying mechanism(s) by which *NICD*-transfected MSC-derived ECM supports neural cell growth and survival, we investigated the differences in *NICD*-transfected MSC- and MSC-derived ECM protein quantity and composition. To compare the ECM derived from MSCs and *NICD*-transfected MSCs, the proteins were sequentially solubilized using sodium dodecyl sulfate (SDS) and urea, quantified, and compared across four human donors. We then analyzed ECM proteins using either in-gel digests or in-solution surfactant-assisted trypsin digests (SAISD) coupled with reverse phase nano-liquid chromatography and tandem mass spectrometry (nLC-MS/MS). Analyses using nLC-MS/MS identified key components of ECM from *NICD*-transfected MSCs and un-transfected MSCs and revealed significant differences in their respective compositions. This work provides a reproducible method for identifying and comparing *in vitro* cell-derived ECM proteins, which is crucial for exploring the mechanisms underlying cellular therapy.

## Introduction

SB623 is a cell therapy product comprised of human bone marrow-derived mesenchymal stromal cells (MSCs) transiently transfected with a *notch1 intracellular domain* (*NICD*)-expressing-plasmid. SB623 is currently in FDA-approved Phase I/IIa clinical testing for ischemic stroke. SB623 has been shown to improve functional behavior deficits and reduce neural cell loss in stroked rats, however governing biological mechanisms remain to be elucidated [1]–[2]. Previous reports have demonstrated that MSCs promote neuroprotection/regeneration without replacing damaged/dead neural cells, but instead offer indirect supporting mechanisms for survival and regeneration in the central nervous system [3]–[4]. SB623 has been previously shown to rescue neural cells via paracrine factors following *in vitro* ischemia [5].

Molecules in the ECM have been shown to aid in the development of the nervous system, including neuronal survival, migration, axonal growth, synapse formation and glial differentiation [6]. The ability of MSC- and SB623-derived ECM to support the growth of rat primary cortical cells has previously been demonstrated [7]. In the same study, SB623-derived ECM was shown to have a greater ability to promote neural cell growth compared to un-transfected MSCs, suggesting differences between these matrices. In the current study, we are comparing quantities and composition of ECM produced by MSC and SB623.

To this end, a non-enzymatic isolation, detergent/chaotropic solublization, chloroform/methanol precipitation, followed by an in-gel digest or surfactant assisted in-solution digest (SAISD) for nano-liquid chromatography tandem-mass spectrometry (nLC-MS/MS) analysis was employed. Over 20 ECM proteins were identified with 11 significant differences in ECM protein expression between SB623 and MSC. These differences may help explain SB623-derived ECM’s greater ability to promote neural cell growth over MSC.

## Methods and Materials

### MSC and SB623 Cell Preparation

Human adult bone marrow aspirates were purchased from Lonza (Walkersville, MD). Cells were washed once and plated in T-225 flasks (Corning, Corning, NY) in α minimal essential medium (αMEM;Mediatech, Herndon, VA) supplemented with 10% fetal bovine serum (FBS; Hyclone, Logan, UT), 2 mM L-glutamine, and penicillin/streptomycin (both from Invitrogen, Carlsbad, CA). After 3 days, unattached cells were removed; the MSC cultures were maintained in the growth medium for about 2 weeks and then subcultured with 0.25% trypsin/EDTA (Invitrogen). On the second passage, some of the cells were cryopreserved (MSC preparation), and the others plated for the preparation of SB623 cells. For this, MSCs were transfected with the pCI-neo expression plasmid encoding human *Notch intracellular domain* (*NICD*). The transfection was performed with Fugene 6 (Roche Diagnostics, Indianapolis, IN) according to the manufacturer’s protocol. On the next day, the medium was replaced with growth medium containing 100 µg/ml G418 (Invitrogen), and the selection continued for 7 days. The selection medium was then replaced with G418-free growth medium. The cultures were maintained for about 2 weeks and twice expanded by subculturing. The resulting SB623 cells were harvested and cryopreserved. The MSCs and SB623 cells were stored in the vapor phase of liquid nitrogen until they were needed. Both the MSCs and SB623 were characterized by flow cytometry on the second passage and before cryopreservation and were found to be positive for CD29, CD90, and CD105 (>95%), and negative for CD31, CD34, and CD45 (<5%), confirming their mesenchymal nature. All experiments described here were performed with MSCs and SB623 cells that were cryopreserved, then thawed, grown for 5–6 days to allow recovery, and plated for the experiments.

### ECM Preparation

**Figure 1:**
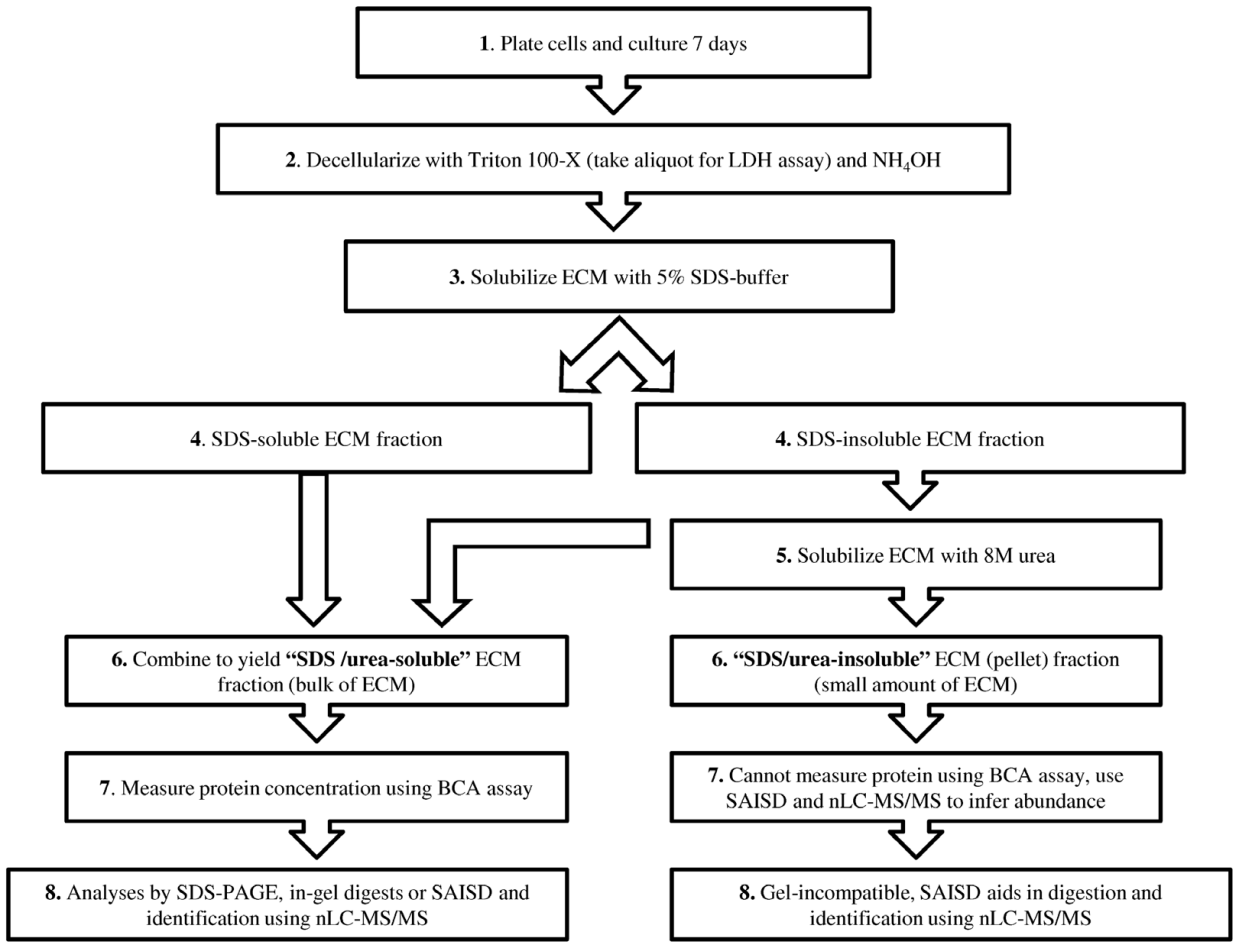
Flow chart of ECM preparation, solubilization and analysis.

For the preparation of cell-produced ECM, either MSCs or SB623 cells were plated at 2.7×10^4^ cells/cm^2^ on 10-cm plates (Nunc, Denmark), in growth medium. After 5 days, the growth medium was changed to serum-free medium, and the cells were cultured for an additional 2 days. The ECM was prepared using a published protocol, with some modifications [8]. Briefly, plates were treated with 5 ml 0.2% Triton X-100 (Sigma-Aldrich, St. Louis, MO) in water at room temperature for 10 min. Cell lysates were aspirated at low suction force, and a 5-ml solution of 0.3% ammonium hydroxide (Sigma-Aldrich) in water was slowly added to the wells for 5 min. The plate was checked under a microscope to ensure full decellularization. Then, the plates were carefully washed with phosphate-buffered saline (PBS), treated with 120 Ku/ml of DNase-I (Sigma-Aldrich) and 2 mM phenylmethylsulfonic fluoride (PMSF;Sigma-Aldrich) for 30 min at room temperature and solubilized immediately. In experiments comparing cell numbers, 200-µl aliquots of MSC and SB623 cell lysates were removed 5 min after the addition of 0.2% Triton X-100 solution and used in a lactate dehydrogenase (LDH) activity assay (see Figure 1 for flow chart).

### LDH Assay

An intracellular LDH activity test was used to quantify relative numbers of viable cells in cultures as described by Allen *et al* (1994). [9]. A 100-µl sample of 0.2% Triton X-100 cell lysates were diluted in 0.2% Triton X-100 and added to each well in a 96-well microplate in duplicates (Corning). Standards were prepared by serial dilutions of bovine LDH (Sigma-Aldrich) in 0.2% Triton X-100 on each plate in duplicates. The activity of intracellular LDH was immediately assayed with 100 µl of catalyst and colorimetric substrate (mixed together for a total of 100 µl) supplied in the LDH kit (Roche Diagnostics, Germany). Colorimetric analysis was performed according to the manufacturer’s protocol with a SpectraMax Plus plate reader (Molecular Devices, Sunnyvale, CA) equipped with SoftMax Pro software. LDH activity results measured in mU/ml, were used to normalize SB623- and MSC derived ECM protein concentrations (BCA assay).

### ECM Collection and Solubilization

After ECM had been prepared and treated with DNase-I, it was washed again with 10 ml PBS and aspirated. The plates were set on a slight angle to allow for PBS to totally drain for 5 min and then aspirated again. To solubilize ECM, previously published protocols were used with modifications [10]–[11]; 200 µl of SDS buffer (5% SDS, 10% glycerol, 60 mM Tris-HCL, pH 6.8; all purchased from Sigma-Aldrich) was added to a plate. The plates were scraped with a cell scraper (Corning) and the SDS-lysate was collected into 1.5-ml mini-centrifuge tubes (Axygen, Union City, CA). Then, the SDS-lysates were boiled at 95°C for 5 min, mixed gently and spun down at 16,000×g for 10 min. Supernatants- called “SDS-soluble” ECM protein – were removed and placed on ice. The protein pellets- called “SDS-insoluble” – were processed further. Ten volumes of urea buffer (8 M urea; Sigma-Aldrich), 4% SDS, 60 mM Tris-HCl, 12.5 EDTA (Gibco) and deionized water) were added to the SDS-insoluble protein pellet. The pellet was pipetted repeatedly to break it apart, let stand for 30 min at room temperature, then spun down at 16,000×g for 5 min. The supernatant was combined with SDS-soluble fraction, called “SDS/urea-soluble”, and stored at −80°C for subsequent in-gel or in-solution digests and nLC-MS/MS analysis. Residual pellet, called “SDS/urea-insoluble”, was digested using SAISD for nLC-MS/MS analysis (see SAISD methods).

### BCA Assay

ECM protein concentration was measured using the Micro BCA Protein Assay Kit (Pierce Biotechnology, Rockford, IL) according to the manufacturer’s protocol. SDS/urea-soluble ECM protein samples were diluted in water 1∶10, 1∶20 and 1∶40 in duplicates to dilute interfering contaminants (Tris, SDS, glycerol and urea). Bovine serum albumin (BSA) standards were serially diluted in a 50/50 mixture of SDS and urea buffers (diluted 1∶20 in deionized water). After 1–2 hours at room temperature, a colorimetric analysis was performed with a SpectraMax Plus plate reader. Protein concentration measurements for SB623- and MSC-derived SDS/urea-soluble ECM were normalized to SB623 and MSC relative cell counts (LDH assay), and expressed as concentration (µg/ml) of SB623 to MSC.

### 1D SDS-PAGE and In-Gel Digests

To concentrate SDS/urea-soluble fractions, 100-µl samples (20–30 µg of protein) were precipitated with chloroform and methanol (Sigma-Aldrich) according to Wessel *et al.* (1984) [12]. Precipitates were dried by decanting and heating at 60°C with lids slightly ajar. The pellet was resuspended in 40 µl Tris-glycine SDS Sample Buffer (Novex Invitrogen) supplemented with 5% 1, 4-Dithiothreitol (DTT; Sigma-Aldrich), heated to 95°C for 10 min and centrifuged at 16,000×g for 1 min. After centrifugation, each sample was loaded into a 1.5 mm 4–20% gradient Tris-Glycine gel (Invitrogen) along with Precision Plus molecular weight markers (Bio-Rad). The samples were electrophoresed using Tris-glycine running buffer (Novex Invitrogen) at 125 V for 2 hours. The gel was stained with Coomassie Brilliant Blue R-250 (MP Biomedicals) for 1 hour and destained overnight in methanol, acetic acid (Sigma-Aldrich) and deionized water at 4°C.

Eight prominent gel bands were excised with a stainless steel scalpel. These bands were cut into 1 mm pieces and transferred into separate 0.5 ml centrifuge tubes. To destain, 300 µl of 25 mM ammonium bicarbonate/50% acetonitrile was added and mixed at 35°C for 20 min; then the buffer was discarded. Reduction of ECM protein was performed by covering gel pieces with 100 µl of 35 mM DTT/50 mM ammonium bicarbonate and incubated at 55°C for 25 min. The reduction buffer was removed, then the gel pieces were washed with 50 mM ammonium bicarbonate (wetting buffer). The wetting buffer was removed from the gel pieces and the ECM protein was then alkylated with 100 µl of 50 mM iodoacetamide/50 mM ammonium bicarbonate and incubated at 30°C in the dark for 1 hour. The newly reduced and alkylated ECM protein located in the gel pieces were washed again with wetting buffer and dried with speed-vac. ECM protein within the gel pieces were then subjected to an in-gel trypsin digestion solution (25 µl) of 10 ng/µl sequencing grade trypsin in 50 mM ammonium bicarbonate. Up to 55 µl of wetting buffer was added as needed to insure gel pieces were fully covered for overnight incubation at 37°C. ECM peptides were extracted from gel pieces with 250 µl of 50% acetonitrile/1% formic acid at 38°C for 40 min. This peptide sample was transferred into a new, 0.5-ml centrifuge tube, dried by speed-vac and resuspended in 15 µl of 0.1% formic acid for nLC-MS/MS analysis. All solvents used were from Thermo-Fisher, sequencing grade trypsin was from Promega and chemicals (DTT, iodoacetamide) were from Sigma-Aldrich. ]. Note, samples analyzed using nLC-MS/MS from in-gel digests and SAISD were from different donors.

### Surfactant Assisted In-Solution Digests (SAISD)

SAISD was used for the preparation of SDS/urea-soluble and insoluble SB623-and MSC-derived ECM for nano-liquid chromatography tandem mass spectrometry (nLC-MS/MS). Methods for preparing ECM for nLC-MS/MS were similar to what has been published by Hansen *et al.* (2009) [13], however ultrasonication was not used. In brief, 500 µg of precipitated SDS/urea-soluble and an unknown amount of SDS/urea-insoluble SB623- and MSC-derived ECM from the same human donor (one SDS/urea-soluble fraction for SB623, Table 1; two fractions for SDS/urea-soluble and two fractions of SDS/urea-insoluble from each MSC- and SB623-derived ECM preparations, Table 2) were resuspended in 50 µl of 0.1% *Rapigest* (Waters) and vortexed. DTT was added to the ECM samples to a final concentration of 5 mM and heated at 60°C for 30 min. The reduced ECM samples were cooled to room temperature and alkylated with iodoacetamideto final concentration of 15 mM, and then incubated in the dark for 30 min at room temperature. ECM samples were then boiled at 100°C for 5 min and cooled to room temperature. Sequencing grade trypsin was added to ECM samples at a concentration of 1.2 µg of trypsin per 100 µg of ECM protein (1.2 µg of trypsin was added to SDS/urea-insoluble fractions despite not having ECM protein concentrations). Samples were digested overnight at 37°C and then triflouroacetic acid (TFA; Sigma-Aldrich) was added to a final concentration of 0.5%. Samples were incubated at 37°C for 45 min and spun down at 16,000×g for 10 min. The peptide solution was transferred to a clean 1.5 ml centrifuge tube for nLC-MS/MS. Note, samples analyzed using nLC-MS/MS from in-gel digests and SAISD were from different donors.

**Table 1:** Proteins detected in SB623-derived ECM using In-Gel Digest and nLC-MS/MS or SAISD and nLC-MS/MS.

Identified SB623-derived ECM proteins	Molecular weight, kDa	Total peptide spectral counts
**In-gel digestion**		
Collagen I, α1 (COL1A1)	139	4
Collagen I, α2 (COL2A2)	129	6
Collagen VI, α1 (COL6A1)	344	5
Fibrillin-1 (FBN1)	312	5
Fibronectin 1, transcript variant 5 (FN1, transcript variant 5)	111	8
Fibronectin, isoform 1 (FN1, isoform 1)	263	133
Perlecan (HSPG2)	469	20
Tenascin-C (TNC)	241	4
**Surfactant assisted in-solution digestion (SAISD)**		
Collagen I, α1 (COL1A1)	139	27
Collagen I, α2 (COL1A2)	129	16
Collagen V, α3 (COL5A3)	172	2
Collagen VI, α1 (COL6A1)	109	5
Collagen VI, 2, Isoform 2C2 (COL6A2, isoform 2C2)	109	2
Collagen VI, α2, Isoform 2C2A (COL6A2, isoform 2C2A)	87	2
Collagen VI, α3, isoform 1 (COL6A3, isoform 1)	344	20
Collagen XII, α1, isoform 2 (COL12A1, isoform 2)	205	3
Elastin microfibril interfacer 1 (EMILIN1)	107	9
Fibrillin-1 (FBN1)	312	40
Fibronectin 1, transcript variant 5 (FN1, transcript variant 5)	111	2
Fibronectin, isoform 1 (FN1, isoform 1)	263	83
Fibronectin, isoform 7 (FN1, isoform 7)	269	2
Fibulin-1, isoform B (FBLN1, isoform B)	77	4
Growth/differentiation factor 15 (GDF-15)	34	2
Immunoglobulin-like and fibronectin type III domain-containingprotein 1 (IGFN1)	384	2
Latent-transforming growth factor beta-binding protein 2 (LTBP2)	195	3
Perlecan (HSPG2)	469	76
Tenascin-C, isoform 1(TNC, isoform 1)	241	15
Thrombospondin-1 (TSP1)	129	9
Transforming growth factor-beta-induced protein ig-h3 (TGFBI)	75	9

SDS/urea-soluble ECM from SB623 was precipitated and proteins separated using SDS-PAGE. Eight of the most prominent high molecular weight gel bands were excised. ECM protein was destained, reduced, alkylated, trypsinized, extracted and individually analyzed with nLC-MS/MS. For SAISD, SB623-derived ECM protein was resuspended in 0.1% w/v ammonium bicarbonate and *Rapigest* surfactant powder. The sample was then reduced, alkylated, trypsinized, and analyzed using nLC-MS/MS. Proteins identified had at least two unique peptides. Note, in-gel and SAISD ECM samples analyzed using nLC-MS/MS were from different donors.

**Table 2:** Percentage of total spectral counts for SB623- and MSC-derived ECM proteins identified using SAISD and LC-MS/MS.

ECM protein	SDS/urea-soluble(% of total spectra)			SDS/urea-insoluble(% of total spectra)		
	SB	MSC	p-Value	SB	MSC	p-Value
Collagen I, α1 (COL1A1)	**4.5**	**2.4**	**0.003** *	1.3	0.8	0.083
Collagen I, α2 (COL1A2)	2.5	1.9	0.198	0.3	0.5	0.153
Collagen VI, α1 (COL6A1)	1.1	1.7	0.228	**0.1**	**0.7**	**0.003** *
Collagen VI, α2 (COL6A2)	0.9	1.2	0.497	N/A	N/A	N/A
Collagen VI, α3 (COL6A3)	4.1	6.8	0.074	**0.2**	**1.9**	**<0.001** *
Collagen XII, α1 (COL12A1)	0.3	0.3	0.574	N/A	N/A	N/A
Elastin (ELN)	0.1	0.3	0.279	1.1	1.3	0.259
Elastin microfibril interfacer 1 (EMILIN1)	2.9	2.5	0.567	3.8	4.3	0.363
Fibrillin-1 (FBN1)	2.6	2.4	0.651	**19.7**	**16.5**	**0.024** *
Fibronectin (FN1)	60.1	60.1	0.996	**64.8**	**61.7**	**0.035** *
Fibulin-1 (FBLN1)	**0.0**	**0.3**	**0.038** *	**0.0**	**0.5**	**0.002** *
Filamin-A (FLNA)	1.7	0.9	0.060	0.4	0.6	0.442
Perlecan (HSPG2)	13.5	11.5	0.132	**2.8**	**4.1**	**0.028** *
Latent-transforming growth factor beta-binding protein 1 (LTBP1)	N/A	N/A	N/A	**1.2**	**0.4**	**0.02** *
Latent-transforming growth factor beta-binding protein 2 (LTBP2)	1.5	0.8	0.125	0.7	0.5	0.120
Microfibrillar-associated protein 2 (MFAP2)	N/A	N/A	N/A	0.3	0.0	0.161
Periostin (POSTN)	0.6	0.2	0.074	0.7	1.2	0.051
Tenascin-C (TNC)	**0.8**	**4.2**	**<0.001** *	**0.2**	**1.5**	**<0.001** *
Transforming growth factor-beta-induced protein ig-h3 (TGFBI)	**0.5**	**1.7**	**0.021** *	**0.3**	**2.0**	**<0.001** *
Transglutaminase 2 (TGM2)	**0.7**	**0.0**	**0.002** *	**0.5**	**0.1**	**0.028** *
Thrombospondin-1 (TSP1)	1.0	0.6	0.352	1.2	1.2	0.988
Versican (VCAN)	0.3	0.0	0.195	0.5	0.0	0.050

Values are expressed as an average percentage of total spectral counts based on eight LC-MS/MS runs.

* indicates significant difference between SB623 and MSC ECM (p-value<0.05).

### Peptide/Protein Identifications by nLC-MS/MS Analysis

Tryptic peptides derived from each sample were analyzed using multiple nano liquid chromatography/electrospray ionization-tandem mass spectrometry (electrospray ionization nLC-MS/MS) analyses to maximize protein identification and to improve the reproducible detection of low abundance proteins [14]–[18]. The samples of tryptic peptides were dissolved with 50–100 µl of 0.1% formic acid/water, and analyzed by electrospray ionization nLC-MS/MS using a Thermo LTQ ion trap mass spectrometer with dual Thermo Surveyor HPLC pump systems (Thermo Fisher, San Jose, CA). A new NanoLC (nLC) C18 column for each cell lysate sample eliminated potential peptide carry-over. Tryptic peptides were subjected to a series electrospray ionization nLC-MS/MS analyses with five different settings of the dynamic exclusion (DE) rule for the number of MS/MS spectra acquired with DE = 2 and an excluded time window of 45 sec, or DE = 1 and an excluded time window of 30, 45, 60 and 90 sec. In addition, three different gas fractionation settings with mass ranges of m/z 400–900, m/z 700–1200, or m/z 1000–1800 with DE = 1 and an excluded time window of 60 sec were employed to analyze the samples. Electrospray ionization nLC-MS/MS analyses were conducted using a nLC reverse phase C18 column (75 µm×130 mm). The mobile phases for the reverse phase chromatography were 0.1% HCOOH/water (mobile phase A) and 0.1% HCOOH in acetonitrile (mobile phase B). A four-step, linear gradient was used for nano-LC separation (5% to 35% B in the first 65 min, followed by 35% to 80% B in the next 10 min, holding at 80% B for 5 min, and returning to 5% B during the final 10 min). The ESI-MS/MS data acquisition was set to collect ion signals from the eluted peptides using an automatic, data-dependent scan procedure in which a cyclical series of three different scan modes (1 full scan, 4 zoom scans, and 4 MS/MS scans of the four most abundant ions) was performed. The full scan mass range was set from m/z 400 to 1800. Since the use of multiple algorithms has been shown to reduce the number of false positive identifications and increase the number of protein identifications [19]–[20], two algorithms [Mascot (v2.3) [21] and X!Tandem (2007.01.01.1) [22]] were used to identify peptides from the resulting MS/MS spectra by searching against the combined human protein database (total 22673 proteins) extracted from SwissProt (v57.14; 2010 February) using taxonomy “homo sapiens” (22670 proteins). Searching parameters were set as follows: parent and fragment ion tolerances of 1.6 and 0.8 Da, respectively; carbamidomethyl (+57 Da) modification of Cys as a fixed modification; deamination (+1 Da) of Asn, and oxidation of Met as variable modifications; trypsin as the protease with a maximum of 2 missed cleavages. Scaffold (Proteome Software) was used to merge and summarize the data obtained from the eight runs of electrospray ionization nLC-MS/MS protein identification analyses for each sample preparation. Protein identifications were based on a minimum detection of 2 peptides with 99% protein identification probability using the algorithm ProteinProphet [23]. Each peptide identified had a minimum peptide identification probability of 95% using the algorithm PeptideProphet [24].

### Quantitative Reverse Transcriptase Polymerase Chain Reaction (qRT-PCR)

**Figure 2.**
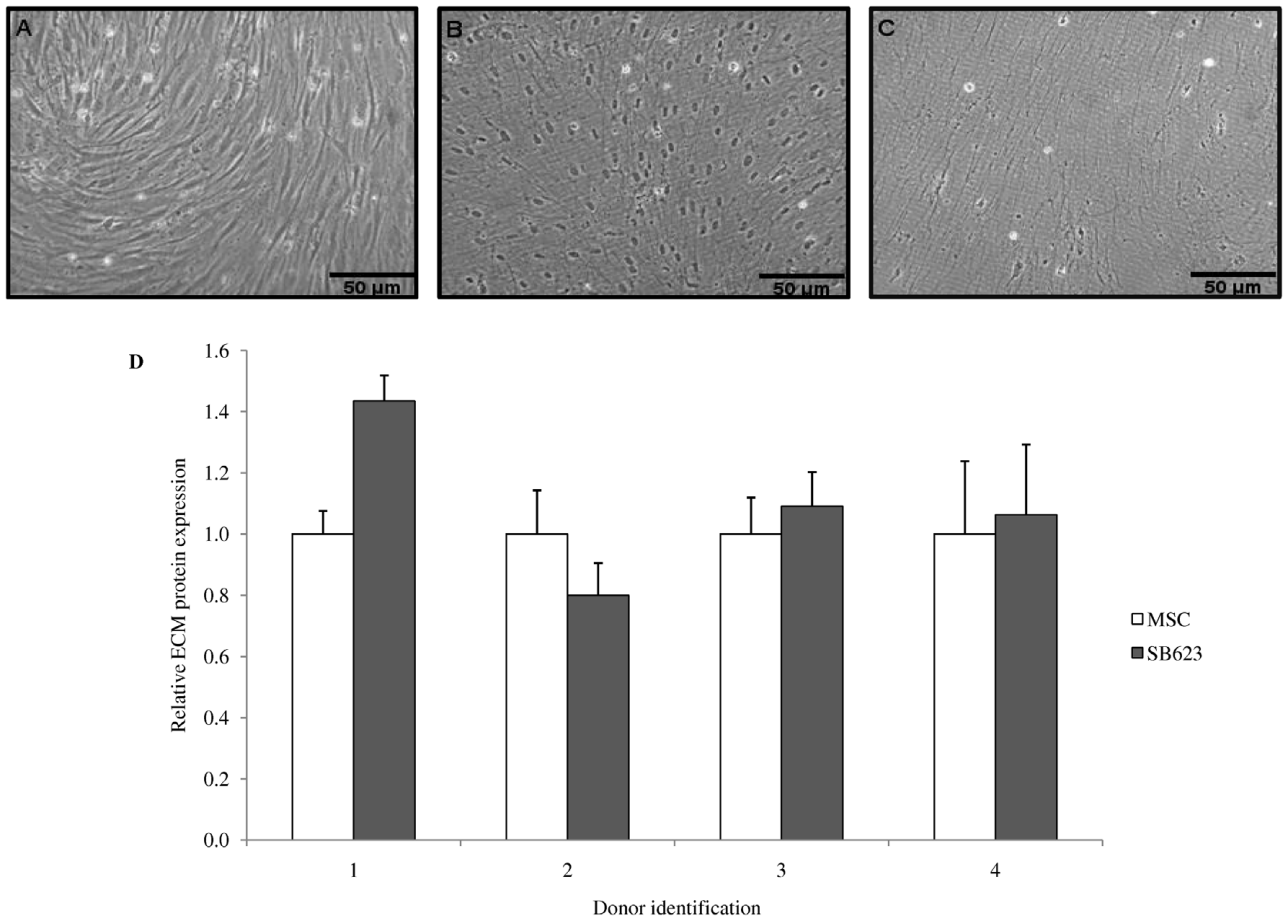
Isolation and relative protein expression comparison of MSC and SB623-derived ECM. SB623 and MSCs were cultured for five days in complete medium followed by two days in serum-free medium (A). Cells were treated with 0.2% Triton X-100 (Triton) (B). Cells were then treated with 0.3% NH_4_OH, DNase I and PMSF, and then rinsed. The isolated ECM has a fibril-matrix like appearance (C). MSCs and SB623 produce similar quantities of ECM proteins. ECM protein concentrations were determined using the BCA assay, and relative ECM protein expression was determined by normalizing to relative cell counts (LDH assay) (D). Scale bar = 50 µm.

For gene expression analysis, cells (1 million) were plated into a 10-cm dish and cultured for 4–5 days. Cells were then lysed and RNA purified using RNeasy kit (Qiagen, Germantown,MD) according to the manufacturer’s protocol. TaqMan gene expression assays (Applied Biosystems/LifeTechnologies) and QuantiTect Probe RT-PCR Master Mix (Qiagen) were used for one-step qRT-PCR. Samples, 5–7 ng RNA per reaction, in duplicates, and standards (prepared from a sample by serially diluting it in water) were analyzed using LightCycler 480 (Roche, Mannheim, Germany), which was programmed according to the Master Mix manufacturer’s protocol, with 40 amplification cycles. The data were analyzed using the second derivative maximum method. Glyceraldehyde 3-phosphate dehydrogenase (GAPDH) expression was used to normalize expression of genes of interest. Data was taken from 5 different human donors, two of which were the same (donors 3 and 4) as in Figure 2, SAISD-nLC-MS/MS donor (Table 2) and two unassociated donors.

### Spectral Counting and Statistical Analysis

To determine if there are differences in production of specific proteins, proteins were identified by nLC-MS/MS and relatively compared using a label-free quantification method referred to as spectral counting (see review, [25]). For this test, corresponding cell samples from 1 human donor were analyzed in 8 independent runs. Spectral counts were normalized for each run as the ratio of total spectral counts for each individual ECM protein to total spectral counts for all ECM proteins, expressed as a percentage and then averaged across each of the 8 runs. For each protein identified, the mean percentages of spectral counts in SB623 and MSC were compared using a t-test. For all analysis, SigmaStat (StyStat Software, San Jose, CA) was used and an alpha value of 0.05 was set to assess if the means were significantly different.

## Results

### ECM Isolation and Relative Quantification Comparison

ECMs were chemically isolated for protein quantification and subsequent analysis. Figure 2A–C shows the sequential steps in decellularization of ECM using 0.2% Triton, 0.3% NH_4_OH, DNase-1, and 2 mM phenylmethanesulfonylfluoride (PMSF). The use of 5% SDS buffer with a subsequent treatment in 8 M urea buffer for protein quantification and ensuing gel-based analysis was chosen because of its ability to solubilize the highest amount of ECM (data not shown).

A comparison of SB623 and MSC ECM protein fractions across four different human bone marrow donors was performed. This comparison addressed whether or not MSC and SB623 produced similar amounts of SDS/urea-soluble ECM. Figure 2D compares the relative amount of ECM expressed by MSC and its derivative, SB623. Donor 1 suggests a difference in protein expression with SB623 secreting 1.4 times that of MSC. However, donors 2–4 did not show marked differences (0.8, 1.1 and 1.1 respectively) in ECM protein expression. When all four donor comparisons are taken into consideration, SB623 and MSC express similar amounts of ECM protein.

**Figure 3:**
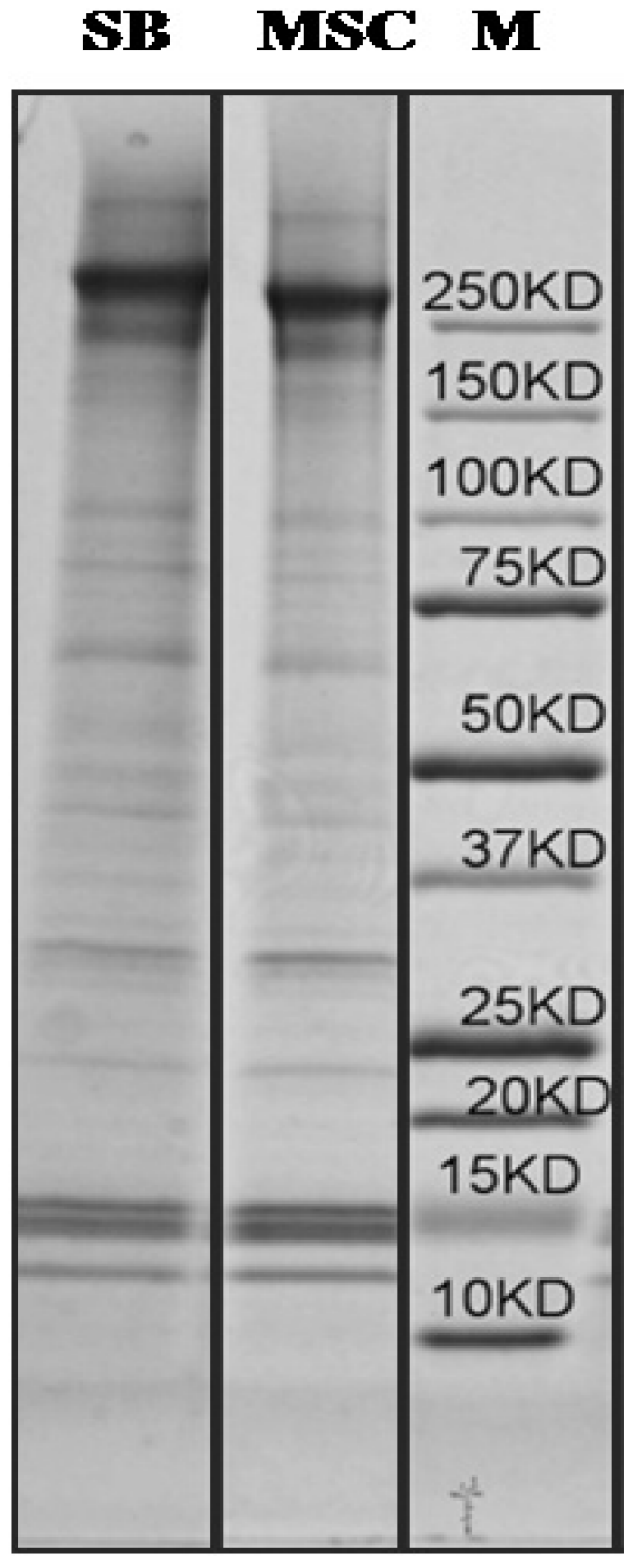
Solubilized MSC and SB623-derived ECM analyzed by SDS-PAGE. SDS/urea-soluble SB623-derived ECM (SB) and corresponding MSC. M: molecular weight markers. SDS/urea samples were precipitated, re-suspended in 2X loading buffer, loaded on a 1.5-mm 4–20% Tris-acetate gel, electrophoresed and stained with Coomassie Blue R-250.

To help elucidate possible compositional differences between SB623 and MSC-derived ECMs SDS-PAGE was utilized. Figure 3 shows SDS-PAGE of chloroform-methanol precipitated SDS/urea-soluble ECM from both SB623 and MSC. Upon comparison of SB623-derived versus MSC-derived ECM there were no obvious differences in band size or distribution. To elucidate possible compositional differences between SB623- and MSC-derived ECMs, protein in each gel band was determined.

### Identification of SB623-derived ECM Proteins using In-Gel Digest and nLC-MS/MS or Surfactant Assisted In-Solution Digest and nLC-MS/MS

**Figure 4:**
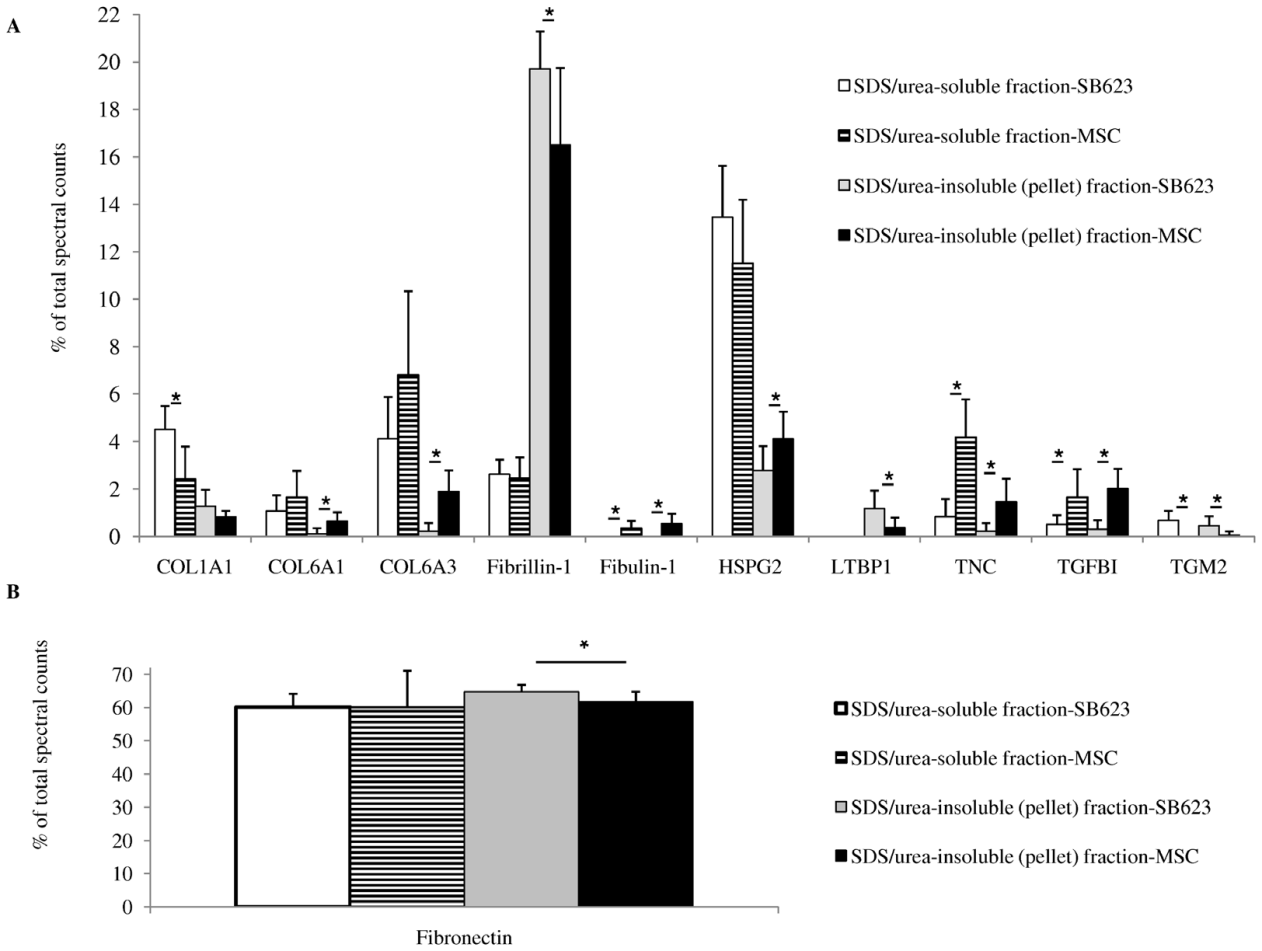
Proteins that exhibit differential expression between SB623- and MSC-derived ECM; either in SDS/urea-soluble or SDS/urea-insoluble fractions or both. Precipitated ECM samples were resuspended in 0.1% w/v ammonium bicarbonate and *Rapigest* surfactant powder. The sample was then reduced, alkylated, trypsinized and analyzed by shotgun nLC-MS/MS. Proteins that were significantly different in abundance between SB623- and corresponding MSC-ECM are plotted in (A), with the exception of fibronectin (FN1), which is plotted in (B) because of its relatively high abundance. Collagen 1 alpha 1 (COL1A1), collagen 6 alpha 1 (COL6A1), collagen 6 alpha 3 (COL6A3), perlecan (HSPG2), latent transforming growth factor binding protein 1 (LTBP1), tenascin-c (TNC), transforming growth factor-beta-induced protein ig-h3 (TGFBI), transglutaminase 2 (TGM2). Mean ± SD; *p-value <0.05.

To identify major components of SB623-derived ECM, eight prominent gel bands from one SDS-PAGE lane (Fig. 4, SB lane) were excised, subjected to an in-gel trypsin digest, and the resulting tryptic peptides were analyzed using nano-liquid chromatography tandem mass spectrometry (nLC-MS/MS;Table 1). The total peptide spectral count is the sum of the MS/MS spectra that matches with the sequence of each protein. Larger total peptide spectral counts represent the more abundant proteins found from all eight excised gel bands. Fibronectin isoform 1 (FN1, isoform 1) and perlecan (HSPG2) showed the highest detection, with 133 and 20 total peptide spectral counts, respectively, while collagen1 (COL1) showed only a total of 10 spectral counts. This sequential method of SDS-PAGE-in-gel trypsin digestion and peptide extraction with nLC-MS/MS allowed for the identification of seven SB623-derived ECM proteins.

While in-gel digests worked for identifying major SB623-derived ECM proteins there is a resistance for certain ECM proteins to run in SDS-PAGE and a difficulty associated with adequately extracting these proteins from gels. SAISD coupled with nLC-MS/MS was chosen as an alternative because it is a gel-free system and allows for more proteins to be detected in a single sample. As seen in the lower part of Table 1, there was a marked increase of peptide detection over in-gel protein digests. For example, tenascin-C (TNC) and HSPG2 displayed 15 and 76 total peptide spectral counts, respectively; a three-fold detection increase over in-gel digest results. More collagen type I α1 and α2 chains were detected using SAISD. Previous detection via in-gel digests resulted in a total of 10 peptide spectral counts of collagen type I (four α1 and six α2 chains), but SAISD yielded 43 peptide spectral counts (27 α1 and 16 α2 chains) showing a four-fold increase in detection. Importantly, the SAISD-based method revealed many ECM proteins that were not identified using in-gel digests including FN1 isoform 7, FN1 transcript variant 5, fibulin-1 (FBLN1), elastin microfibril interfacer 1 (EMILIN-1), immunoglobulin-like and fibronectin type III domain-containing protein 1 (IGFN1), thrombospondin-1 (TSP1), collagen type V, VI and XII (COL5, 6 and 12), transforming growth factor-beta-induced protein ig-h3 (TGFBI), latent transforming growth factor-beta-binding protein-2 (LTBP2) and growth differentiation factor 15 (GDF15). The identification of SB623-derived ECM using SAISD and nLC-MS/MS introduced a reliable and unbiased method for comparing MSC versus SB623-derived ECM.

### Comparison of SB623- and MSC-derived ECM Expression using SAISD and Label-free nLC-MS/MS and qRT-PCR

SAISD coupled with nLC-MS/MS was used to relatively compare ECM proteins produced by SB623 to those produced by corresponding MSCs. In this analysis, SB623-and MSC-derived ECM samples from one donor were tested in 8 independent nLC-MS/MS runs using a label-free spectral counting approach and normalization. The combined raw spectral count data from all nLC-MS/MS runs (not normalized) are shown in Table S1. Before the use of nLC-MS/MS, the ECM was first solubilized with SDS/urea (SDS/urea-soluble fraction). The remaining small pellet (SDS/urea-insoluble fraction) and SDS/urea-soluble fractions were then prepared for nLC-MS/MS using SAISD. This analysis revealed 22 ECM proteins: 7 out of the 22 were newly identified: elastin (ELN), filamin-A (FLNA), latent-TGFβ-binding protein 1 (LTBP1), Microfibrillar-associated protein 2 (MFAP2), periostin (POSTN), versican (VCAN) and transglutaminase 2 (TGM2). Collagen VI, α2 (COL6A2) and Collagen XII, α1 (COL12A1) were found exclusively in the SDS/urea-soluble fraction where LTBP1 and MFAP2 were exclusively found in the SDS/urea-insoluble fraction. Fibrillin-1 (FBN1) was not excusive to the SDS/urea-insoluble fraction, however markedly more FBN1 was detected in this fraction indicating FBN1’s, along with LTBP1’s and MFAP2’s, resistance to solubilize in the SDS/urea buffer. A complete comparative list of ECM proteins found in SB623 and/or MSCs is shown in Table 2.

Figure 4A shows the ECM components that were represented differently between SB623 and MSC in either the SDS/urea-soluble fraction, the SDS/urea-insoluble fraction, or both. Fibronectin spectral counts (Figure 4B) were highly abundant compared to the other proteins and therefore are shown separately.

COL1A1 displayed differential expression in the SDS/urea-soluble fraction, where COL6A1, COL6A3, HSPG2 and LTBP1 were significantly different in the SDS/urea-insoluble portion. TNC, TGFBI, fibulin-1 (FBLN1) and TGM2 all showed significant differences in both SDS/urea-soluble and –insoluble (pellet) fractions indicating a clear difference between SB623- and MSC- ECM composition for this donor.

**Figure 5:**
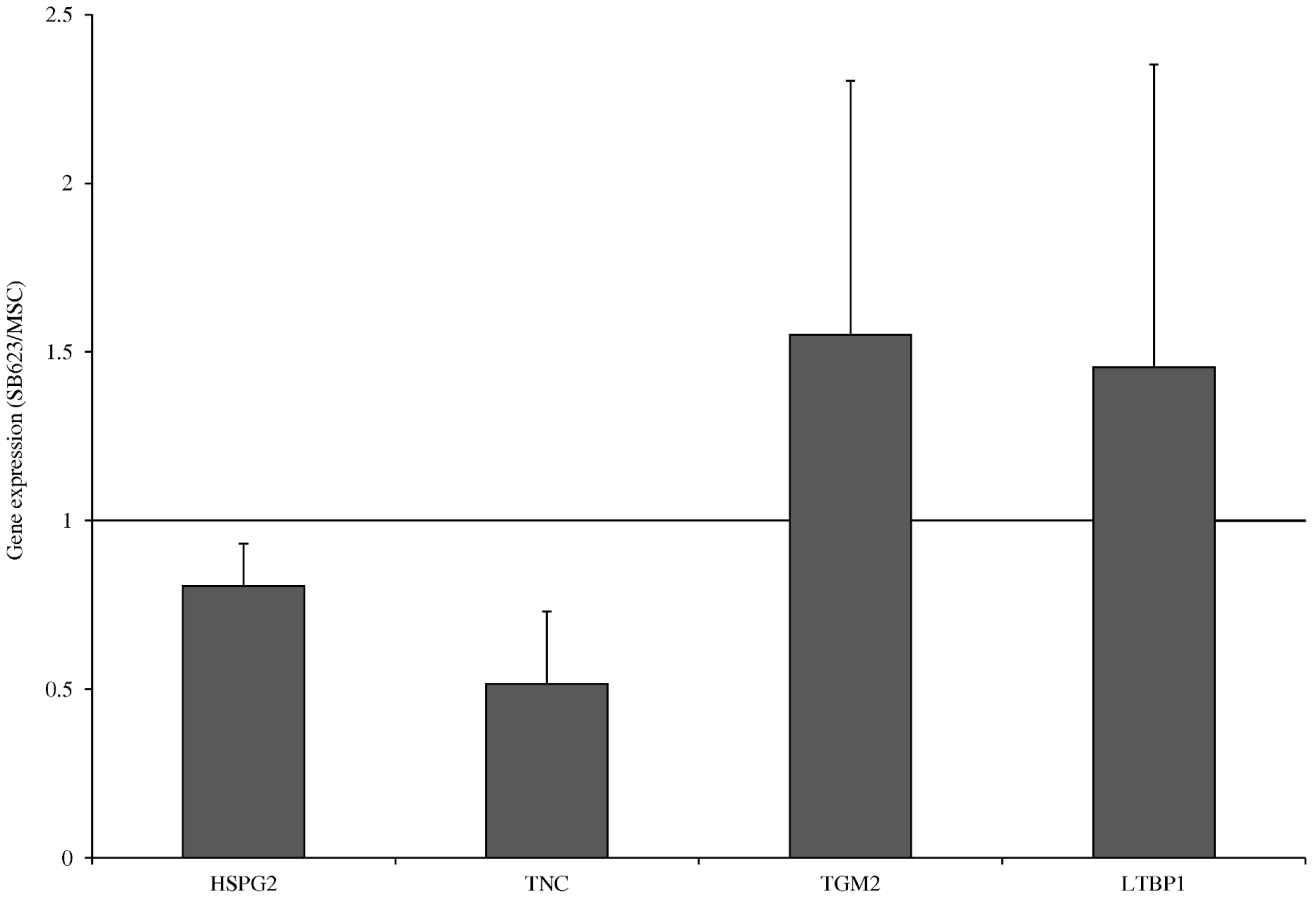
Differences in gene expression levels between SB623 and MSC are consistent with differences detected using SAISD-nLC-MS/MS. Gene expression levels were quantified using qRT-PCR and normalized to the expression of GAPDH; normalized values for MSC were set to 1 and values for SB623 were expressed relative to MSC. Four proteins were analyzed: perlecan (HSPG2), tenascin-C (TNC), transglutaminase 2 (TGM2), and latent-transforming growth factor beta-binding protein 1 (LTBP1). The qRT-PCR data was taken from 5 different human donor pairs, two of which were the same (donors 3 and 4) as in Figure 2, one SAISD-nLC-MS/MS donor (Table 2) and two unassociated donors. Mean ± SD.

To confirm nLC-MS/MS findings and to look for common trends amongst different donors, we used quantitative reverse transcription-polymerase chain reaction (qRT-PCR) to compare mRNA expression levels of four proteins: TGM2, HSPG2, TNC and LTBP1 from five SB623 and MSC donor pairs. As shown in Figure 5, relative mRNA expression levels correlated with findings obtained using nLC-MS/MS (Figure 4A). Relative SB623 TNC and HSPG2 mRNA expression levels were both lower, and TGM2 and LTBP1 mRNA expression levels were both higher when compared to MSC; which is all in agreement with the comparative analysis using nLC-MS/MS.

## Discussion

Researchers have described the role of ECM in the development of the nervous system, including neuronal survival, migration, axonal growth, synapse formation, and glial differentiation [6]. SB623-derived ECM promotes the growth neural cells in culture more efficiently than does MSC-derived ECM [7], but the identification of the composition of this complex matrix had remained elusive. In this study, we implemented methods to solubilize ECM proteins. Further, we established a system that successfully identified and relatively quantified ECM proteins allowing for comparison of ECM derived from MSC and their *NICD*-transfected derivative, SB623.

Literature describing the isolation of an *in vitro* cell-produced ECM and its proteomic analysis is limited (see review, [26]). Optimizing an *in vitro* method for decellularization of SB623 cell- and MSC-derived ECM involved many considerations. Methods using Triton and NH_4_OH dissolve cell/nuclear membranes yielding an intact ECM but allow for intracellular impurities. Cation chelating agents have been reported by other groups to remove monolayers of MSC [27], but from our observations SB623 and MSCs do not fully detach under the same conditions. For this reason the use of Triton and NH_4_OH were used for decellularization, despite that this treatment results in many Triton-soluble intracellular/nuclear proteins that contaminate the ECM (see Table S1).

The ECM contains fibrous proteins such as collagen, fibrillin and fibronectin, which are cross-linked, rendering them insoluble. A two-step ECM solubilization method that included the addition of 5% SDS and 8 M urea was employed and ECM solubilization was confirmed by SDS-PAGE. This allowed for a comparison of SB623- and MSC-derived SDS/urea-soluble ECM production using a classic protein quantification method (BCA assay), which revealed no systematic differences for 4 distinct donors, suggesting that transient transfection of MSC with *NICD* has negligible effects on the overall quantity of SDS/urea-soluble ECM produced. The lack of systematic differences in SDS/urea-soluble ECM protein production prompted a deeper evaluation of SB623- and MSC-derived ECMs composition.

Before thoroughly comparing MSC- and SB623-derived ECM, an experiment was performed to identify major components of SB623-derived ECM utilizing SDS-PAGE, in-gel digests, and nLC-MS/MS. This method identified seven SB623-derived ECM proteins: perlecan (HSPG2), tenascin-C (TNC), fibronectin1 (FN1), collagen type I alpha 1 and 2 (COL1A1/A2), collagen type VI alpha 1 (COL6A1) and fibrillin1 (FBN1). To further optimize identification of ECM proteins and to avoid problems associated with fibrous proteins running in gels, ECM proteins were digested by SAISD then analyzed using nLC-MS/MS. Not using gels affords the opportunity to detect fibrous proteins but also allows for more protein to be analyzed at once. At first glance it appeared FN1 was detected less with SAISD showing 83 total spectral counts and 133 total spectral counts for in-gel digests. This is likely due to the fact that FN1 is more denatured in the presence of SDS-PAGE which allows better trypsin digestion. SAISD-nLC-MS/MS revealed 20 ECM proteins, 12 of which were not identified using in-gel digests including; FN1 isoform 7, FN1 transcript variant 5, fibulin-1 (FBLN1), elastin microfibril interfacer 1 (EMILIN-1), immunoglobulin-like and fibronectin type III domain-containing protein 1 (IGFN1), thrombospondin-1 (TSP1), collagen type V, VI and XII (COL5, 6 and 12), transforming growth factor-beta-induced protein ig-h3 (TGFBI), latent transforming growth factor-beta-binding protein-2 (LTBP2) and growth differentiation factor 15 (GDF15). Other groups researching ECM produced by MSCs, have confirmed by indirect methods, such as immunocytochemistry, the production of ECM proteins found in our analyses. Some of these ECM proteins include, but are not limited to, COL1, HSPG2, FN1 and versican (VCAN) [28]–[30]. Nearly all the ECM proteins found in this analysis have been listed in a contemporary review describing proteomic methods to identify ECM proteins from various cell types [26].

Next, an in-depth comparison of MSC- and SB623-derived ECM was made for one donor. Contrasting MSC and SB623 SDS/urea-soluble and -insoluble ECM using nLC-MS/MS and label-free spectral counting revealed 22 ECM proteins produced by either SB623 or MSC or both. Eleven of these 22 were expressed significantly different between SB623 and MSC: COL1A1, COL6A1, COL6A3, fibrillin-1 (FBN1), fibulin-1 (FBLN-1), HSPG2, LTBP1, and FN1 were significantly different in either the SDS/urea-soluble or SDS/urea-insoluble fraction. TNC, TGFBI, and TGM2 were significantly different in both SDS/urea-soluble and the SDS/urea- insoluble fractions when comparing SB623- and MSC-derived ECM. While some of the differences in ECM composition between SB623 and MSC may be influenced by cell passage number (data not shown), the determination of which genes are affected by passage number or transfection with *NICD* will require further experimentation. Differences between MSC and SB623 observed in nLC-MS/MS data correlated to gene expression levels for TGM2, HSPG2, LTBP1, and TNC. Interestingly there were a group of ECM proteins that were detected by nLC-MS/MS at much higher rates in the SDS/urea-insoluble fraction than that of the soluble fraction. These proteins include elastin (ELN), thrombospondin-1 (TSP1), periostin (POSTN), LTBP1, FN1, FBN1, microfibrillar-associated protein 2 (MFAP2) and elastin microfibril interfacer 1 (EMILIN1) (see Table S1 for spectral counts). These eight proteins are known to have specific interactions with each other and assemble into insoluble microfibrils [31]–[33]. Finding a large majority of these microfibrilary components in the SDS/urea-insoluble fraction elucidates what components one might miss when performing analyses on SDS/urea-soluble ECM proteins only.

Of the 22 proteins of interest, five have been reported to promote neuroregeneration: TNC, HSPG2, thrombospondin-1 (TSP1), FN1, and growth and differentiation factor-15 (GDF-15). Rigato *et al.* (2002) [34] have shown TNC to promote neurite outgrowth modulated through its fibronectin type III BD domains. TNC has been also been implicated in hippocampal-based learning and synaptic plasticity [35]. Yu *et al.* (2011) [36] showed TNC involvement in locomotor recovery after spinal cord injury. HSPG2 has been described by Lee *et al.* (2010) [37] to be pro-angiogenic and neuroprotective after ischemic stroke in rats. FN1 has been associated promotion of angiogenesis, neural crest cell migration and is neuroprotective in stroke and traumatic brain injury [38]–[41]. TSP-1 is a key regulator of synaptogenesis and secreted by astrocytes in the central nervous system [42]–[43]. Finally, GDF-15 was found to promote angiogenesis in hypoxic human umbilical vein endothelial cells [44] and to be a novel trophic factor for midbrain dopaminergic neurons [45]. For the donor we tested, TNC and HSPG2 were down-regulated in SB623, FN1 was up-regulated, and there were no differences in deposition of TSP-1 when comparing to MSC. Future comparison across multiple donors will reveal whether these trends are typical of SB623.

Fibrillin-1, LTBP-1, FN1, and transglutaminase 2 (TGM2) were significantly up-regulated, and fibulin-1 was down-regulated in SB623-derived ECM compared to MSC-derived ECM. These five proteins have been shown to play an interesting and important role in latent TGFβ localization in the ECM. TGFβ has been shown to be neuroprotective in the central nervous system and is induced after ischemic stroke [46]–[48]. Schwann cells promote synaptogenesis via TGF-β1 in the neuromuscular junction [49]. LTBPs covalently bound to TGFβ can bind fibrillin-1 and also be cross-linked to other unknown ECM proteins via TGM2. Fibulin is known to compete with LTBP for fibrillin-1, resulting in more soluble or released LTBP-latent TGFβ complex in the extracellular milieu [31]–[33], [50]–[52]. Thrombospondin-1 induces the activation of latent TGFβ [53]. All together, the properties of these ECM molecules may play a role in localization and activation of latent TGFβ. Additionally, fibrillin-1 has been shown to be a docking site for BMP-2, 4, and 7, all of which are important in the development of the brain [54].

### Conclusion

Thus, we have identified several proteins that may account for the enhanced efficacy of SB623-derived ECM compared to MSC-derived ECM in providing support for neural cell growth. More importantly, we have introduced a reproducible method for identification and comparison of *in-vitro* cell-derived ECM proteins, which is important to better understand mechanisms underlying the therapeutic effects of SB623 cells and other cellular therapies.

## Supporting Information

Table S1 **Total raw spectral counts for SB623- and MSC-derived ECM proteins identified using SAISD and LC-MS/MS.**
(DOCX)
