# Supplementary material for: Proteomic Analysis of the Extracellular Matrix Produced by Mesenchymal Stromal Cells: Implications for Cell Therapy Mechanism
Source: PLoS One. 2013 Nov 14;8(11):e79283. doi: 10.1371/journal.pone.0079283 (PMC3828366; doi:10.1371/journal.pone.0079283)
Supplement: Table S1 — Total raw spectral counts for SB623- and MSC-derived ECM proteins identified using SAISD and LC-MS/MS. (DOCX) [file pone.0079283.s001.docx]

**Table S1**

| **Protein Name** | **MW (kDa)** | **SB623 SDS/urea-soluble** | **MSC SDS/urea-soluble** | **SB623 SDS/urea-insoluble** | **MSC SDS/urea insoluble** |
| --- | --- | --- | --- | --- | --- |
| 5'-nucleotidase (NT5E) | 63 | 16 | 4 | 0 | 0 |
| 78 kDa glucose-regulated protein (HSPA5) | 72 | 7 | 0 | 0 | 0 |
| Actin, alpha cardiac muscle 1 (ACTC1) | 42 | 7 | 0 | 2 | 3 |
| Actin, cytoplasmic 1 (ACTB) | 42 | 60 | 33 | 51 | 56 |
| Alpha-actinin-1 (ACTN1) | 103 | 0 | 0 | 2 | 0 |
| Apolipoprotein B-100 (APOB) | 516 | 0 | 10 | 1 | 4 |
| Basement membrane-specific heparan sulfate proteoglycan core protein (HSPG2) | 469 | 196 | 149 | 48 | 83 |
| Collagen alpha-1(I) chain (COL1A1) | 139 | 68 | 31 | 23 | 17 |
| Collagen alpha-1(VI) chain (COL6A1) | 109 | 16 | 23 | 2 | 12 |
| Collagen alpha-1(XII) chain (COL12A1) | 333 | 3 | 4 | 0 | 1 |
| Collagen alpha-2(I) chain (COL1A2) | 129 | 36 | 23 | 6 | 11 |
| Collagen alpha-2(VI) chain (COL6A2) | 109 | 10 | 15 | 0 | 0 |
| Collagen alpha-3(VI) chain (COL6A3) | 344 | 59 | 92 | 3 | 35 |
| Core histone macro-H2A.1 (H2AFY) | 40 | 12 | 0 | 0 | 0 |
| Cytoskeleton-associated protein 4 (CKAP4) | 66 | 17 | 5 | 0 | 0 |
| Dolichyl-diphosphooligosaccharide--protein glycosyltransferase subunit 1 (RPN1) | 69 | 2 | 0 | 0 | 0 |
| Elastin (ELN) | 68 | 1 | 5 | 18 | 26 |
| Elongation factor 1-alpha 1 (EF1A1) | 50 | 0 | 0 | 8 | 9 |
| EMILIN-1 (EMILIN1) | 107 | 41 | 35 | 69 | 94 |
| Fibrillin-1 (FBN1) | 312 | 36 | 31 | 361 | 332 |
| Fibronectin (FN1) | 263 | 882 | 712 | 1184 | 1250 |
| Fibulin-1 (FBLN1) | 77 | 0 | 5 | 0 | 11 |
| Filamin-A (FLNA) | 281 | 22 | 11 | 7 | 11 |
| Glia-derived nexin (SERPINE2) | 44 | 1 | 1 | 1 | 1 |
| Glyceraldehyde-3-phosphate dehydrogenase (GAPDH) | 36 | 5 | 4 | 17 | 4 |
| Heat shock cognate 71 kDa protein (HSPA8) | 71 | 3 | 0 | 0 | 4 |
| Hemoglobin subunit alpha (HBA1) | 15 | 1 | 1 | 1 | 7 |
| Histone H1.2 (HIST1H1C) | 21 | 6 | 4 | 0 | 0 |
| Histone H2A type 1-B/E (HIST1H2AB) | 14 | 19 | 2 | 15 | 7 |
| Histone H2B type 1-D (HIST1H2BD) | 14 | 16 | 3 | 6 | 1 |
| Histone H3.1 (HIST1H3A) | 15 | 9 | 0 | 2 | 0 |
| Histone H4 (HIST1H4A) | 11 | 26 | 3 | 6 | 0 |
| Keratin, type II cytoskeletal 1 (KRT1) | 66 | 3 | 5 | 1 | 2 |
| Lamin-A/C (LMNA) | 74 | 24 | 11 | 4 | 4 |
| Latent-transforming growth factor beta-binding protein 1 (LTBP1) | 187 | 2 | 0 | 23 | 8 |
| Latent-transforming growth factor beta-binding protein 2 (LTBP2) | 195 | 22 | 8 | 13 | 9 |
| Microfibrillar-associated protein 2 (MFAP2) | 21 | 0 | 0 | 5 | 1 |
| Mitochondrial inner membrane protein (IMMT) | 84 | 4 | 5 | 0 | 0 |
| Myosin light polypeptide 6 (MYL6) | 17 | 6 | 1 | 5 | 2 |
| Myosin-9 (MYH9) | 227 | 86 | 38 | 109 | 77 |
| Myosin-Ic (MYO1C) | 122 | 1 | 2 | 1 | 0 |
| Periostin (POSTN) | 93 | 8 | 4 | 12 | 21 |
| Plectin-1 (PLEC1) | 532 | 2 | 0 | 1 | 0 |
| Protein-glutamine gamma-glutamyltransferase 2 (TGM2) | 77 | 10 | 0 | 8 | 1 |
| Prothrombin (F2) | 70 | 0 | 4 | 6 | 6 |
| Pyruvate kinase isozymes M1/M2 (PKM2) | 58 | 4 | 3 | 12 | 10 |
| Serpin H1 (SERPINH1) | 46 | 7 | 2 | 3 | 1 |
| Talin-1 (TLN1) | 270 | 0 | 0 | 3 | 5 |
| Tenascin (TNC) | 241 | 12 | 52 | 3 | 26 |
| Thrombospondin-1 (TSP1) | 129 | 12 | 6 | 18 | 22 |
| Thy-1 membrane glycoprotein (THY1) | 18 | 5 | 1 | 0 | 2 |
| Transforming growth factor-beta-induced protein ig-h3 (TGFBI) | 75 | 8 | 24 | 5 | 41 |
| Tubulin alpha-1B chain (TUBA1B) | 50 | 8 | 6 | 18 | 19 |
| Tubulin beta chain (TUBB) | 50 | 0 | 0 | 10 | 9 |
| Versican core protein (VCAN) | 373 | 4 | 1 | 10 | 1 |
| Vimentin (VIM) | 54 | 31 | 24 | 12 | 17 |
| Voltage-dependent anion-selective channel protein 1 (VDAC1) | 31 | 19 | 8 | 0 | 0 |
| Voltage-dependent anion-selective channel protein 2 (VDAC2) | 32 | 11 | 8 | 0 | 0 |
